# Marine coastal sediments microbial hydrocarbon degradation processes: contribution of experimental ecology in the omics’era

**DOI:** 10.3389/fmicb.2014.00039

**Published:** 2014-02-12

**Authors:** Cristiana Cravo-Laureau, Robert Duran

**Affiliations:** Equipe Environnement et Microbiologie UMR IPREM 5254, Université de Pau et des Pays de l’AdourPau, France

**Keywords:** microcosm, mesocosm, molecular ecology, intertidal sediments, experimental ecology, omic’s approaches

## Abstract

Coastal marine sediments, where important biological processes take place, supply essential ecosystem services. By their location, such ecosystems are particularly exposed to human activities as evidenced by the recent Deepwater Horizon disaster. This catastrophe revealed the importance to better understand the microbial processes involved on hydrocarbon degradation in marine sediments raising strong interests of the scientific community. During the last decade, several studies have shown the key role played by microorganisms in determining the fate of hydrocarbons in oil-polluted sediments but only few have taken into consideration the whole sediment’s complexity. Marine coastal sediment ecosystems are characterized by remarkable heterogeneity, owning high biodiversity and are subjected to fluctuations in environmental conditions, especially to important oxygen oscillations due to tides. Thus, for understanding the fate of hydrocarbons in such environments, it is crucial to study microbial activities, taking into account sediment characteristics, physical-chemical factors (electron acceptors, temperature), nutrients, co-metabolites availability as well as sediment’s reworking due to bioturbation activities. Key information could be collected from *in situ* studies, which provide an overview of microbial processes, but it is difficult to integrate all parameters involved. Microcosm experiments allow to dissect in-depth some mechanisms involved in hydrocarbon degradation but exclude environmental complexity. To overcome these lacks, strategies have been developed, by creating experiments as close as possible to environmental conditions, for studying natural microbial communities subjected to oil pollution. We present here a review of these approaches, their results and limitation, as well as the promising future of applying “omics” approaches to characterize in-depth microbial communities and metabolic networks involved in hydrocarbon degradation. In addition, we present the main conclusions of our studies in this field.

## INTRODUCTION

**FIGURE 1:**
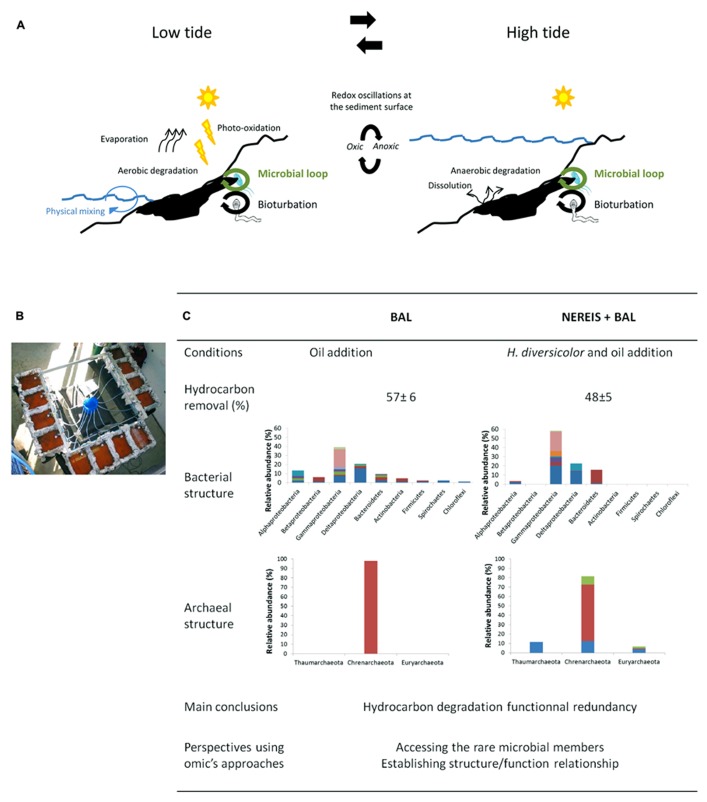
**Mimicking environmental conditions in experimental ecology.** **(A)** Schematic overview of the main abiotic and biotic mechanisms determining the fate of hydrocarbons in coastal marine sediments according to the tide level. At the intertidal zone, where redox conditions oscillate at the sediments’ surface, microbial communities alternate aerobic and anaerobic hydrocarbon degradation processes. Bioturbation processes by macrofauna include sediments reworking, oil burying, and micro-scale oxygen oscillations into the sediments. The microbial loop is controlled by top-down (predation by grazers and viruses) and bottom-top (nutrients and carbon fluxes) processes. **(B)** Experimental device used to assess the effect of crude oil on bio-turbated intertidal marine sediments applying tide cycles. **(C)** Main results and conclusions comparing oil polluted sediments with (NEREIS+BAL) and without *H. diversicolor *addition (BAL) from Stauffert et al. (2013a), 2013b. Bacterial and archaeal communities structures are based on sequences data from 16S rRNA gene transcripts libraries. Colors indicate OTU diversity within each phylum.

Since Amoco Cadiz oil spill in 1978, many other oil spills such as Exxon Valdez (1989), Erika (1999), Prestige (2002), Deepwater Horizon (DH, 2010) have occurred in marine ecosystems. Such oil spill catastrophes generate a lot of indignation among the human populations, especially in coastal areas where environmental injuries are obvious. Cleanup efforts are urgently implemented in order to mitigate the toxic impact of petroleum compounds on the environment. However, the complete recovery of the ecosystem functioning is difficult to achieve because our knowledge on microbial communities, main actors involved in biodegradation processes, is still limited. The main issues regarding marine oil pollution have been already discussed (for review, see McGenity et al., 2012), and microbial processes involved on hydrocarbon degradation extensively described (Timmis et al., 2010; McGenity, 2014). However coastal marine sediments constitute particular ecosystems, especially intertidal zones where environmental conditions are daily modified according to tide level that in turn drive microbial degradation processes (**Figure 1A**). Although the impact of petroleum on microbial communities resulting in ecological succession, modifications of microbial populations following the hydrocarbon degradation, has been largely demonstrated (Harayama et al., 2004; Bordenave et al., 2004, 2007; Head et al., 2006; Païssé et al., 2008; Nogales et al., 2011) several scientific ecological questions remains to be solved. Among these questions, the organization of microbial community structures facing the presence of spilled oil, the mechanisms involved in their adaptation conducting to efficient hydrocarbon degradation, the structure/function relationship and the contribution of functional redundancy to microbial community resilience are some of the current burning questions which responses, at the applied point of view, will help to conduct appropriate bioremediation strategies such as bio-augmentation and bio-stimulation. The presence of a pollutant, such as petroleum, in the environment highlights also the importance to address academic concerns in microbial ecology, contributing more generally to the ecological theory. The importance to apply theory in microbial ecology has been emphasized by Prosser et al. (2007), especially for addressing population ecology, micro-organisms interactions, community assembly and the biodiversity-function relationships. Different approaches including *in situ* studies and laboratory experiments have been developed in order to test ecological hypothesis, decipher the role of microbial communities in the ecosystem functioning and understand the microbial behavior in front of a pollution. Microbial experimental systems have been particularly useful to address ecological questions by simplifying microbial systems and allowing experimental controls (see reviews by Jessup et al., 2004, 2005). Combined with the recent advances in meta-omics’ technologies that provides powerful tools for analyzing microbial communities, their diversity and their functioning as a whole (for review, see Röling et al., 2010; Liu et al., 2013), such experimental microbial systems are promising approaches to gain new insights on functional networks involved in hydrocarbon degradation processes in marine coastal sediments. We review here the recent progress on the ecology of microbial communities involved on hydrocarbon degradation in marine coastal sediments, attained by both *in situ* and experimental approaches. We present the main conclusions on our work in this field indicating the convenience of using experimental ecology to improve our knowledge in hydrocarbon microbial ecology.

### *IN SITU* MICROBIAL ECOLOGY IN COASTAL MARINE POLLUTED SEDIMENTS

Opening the microbial black box involved in hydrocarbon degradation needs to take into consideration the whole ecosystem. *In situ* studies allow to reach the entire microbial communities in their actual context, considering (biotic and abiotic) ecological interactions. Over the past few years, field studies have been performed in marine sediments addressing the impact of oil on microbial communities, by following their organization and/or by characterizing their composition. In most cases this has been approached by spatial comparisons of contrasting contaminated and uncontaminated sites (El-Tarabily, 2002; Miralles et al., 2007, 2010); or sites with different oil contents (LaMontagne et al., 2004; Dias et al., 2011; Iannelli et al., 2012; Kimes et al., 2013). In this way, following the bacterial diversity of oil-polluted retention basin sediments from the Berre lagoon (France) through nine stations, we have demonstrated that bacterial community structure was associated with the gradient of oil contamination (Païssé et al., 2008). Nevertheless, the adaptation of the bacterial community to oil contamination was not characterized by the dominance of oil-degrading bacteria, but a predominance of bacterial populations associated to the sulfur cycle was observed. Other *in situ* studies have highlighted the presence or the role of sulfur cycle microorganisms in oil-polluted coastal marine sediments, with a focus on sulfate-reducing bacteria (Rosano-Hernandez et al., 2012; Acosta-González et al., 2013). Andrade et al. (2012) suggested that sulfate-reducers constitute a large fraction of the bacteria present in oil-contaminated mangrove sediment. They also demonstrated that abundance of sulfate-reducers decrease with depth and showed greater bacterial abundance and diversity in top layers (0–5 cm) than in deeper layers (below 15 cm). Similarly, Acosta-González et al. (2013), applying culture-dependent and molecular techniques to characterize the bacterial populations after the Prestige oil spill at two sampling times (2004 and 2007), reported the dominance of sulfate-reducing bacteria in oil-polluted sediments. Sulfate reduction was the predominant type of respiration connected to hydrocarbon oxidative capacities, and sulfate-reducing bacteria constituted the prevalent populations (being maximal at 12–15 cm depth). These studies emphasized the ecological importance of sulfate-reducers in oil-polluted marine sediments. Sulfate-reducing microorganisms are known to play a key role in coastal marine ecosystems by recycling the organic matter (Jorgensen, 1982), even after an oil-spill. Acosta-González et al. (2013) also demonstrated that community structure was initially dominated by Gamma and Deltaproteobacteria (2004), while 3 years late, in 2007, the phylum Bacteroidetes was a main component of the community. These results, showing the great plasticity of bacterial community structures and suggesting that they were constantly adapting to the changing environmental factors, highlight the importance to consider the impact of environmental parameters on microorganisms when studying oil degradation in coastal marine sediments. In this way Kostka et al. (2011), considering spatial-temporal variations in oil-contaminated beach ecosystems, showed the selective response of bacterial communities to oil from the DH oil spill. Microbial communities were dominated by members of the Gammaproteobacteria and Alphaproteobacteria, suggested as key players in oil-degradation. These groups are constituted by hydrocarbon-degrading members, the former contributing to the early stages of oil hydrocarbon degradation (oxidizing more reactive components such as *n*-alkanes), and the latter contributing to the later stages of degradation (oxidizing more recalcitrant compounds such as PAHs). In the same way, examining microbial response to DH oil spill in coastal sediments, Horel et al. (2012) showed no seasonal differences in the abundances of total hydrocarbon and alkane degraders in marsh ecosystem with high physical–chemical parameters variations.

Different molecular approaches, mainly involving 16S rRNA gene analyses such as DGGE (LaMontagne et al., 2004; Al-Thukair et al., 2007; Dias et al., 2011; Andrade et al., 2012; Isaac et al., 2013) or T-RFLP (Edlund and Jansson, 2006; Païssé et al., 2008; Iannelli et al., 2012), clone libraries (LaMontagne et al., 2004; Miralles et al., 2007, 2010), and phylo/geochip (Beazley et al., 2012), were used to describe the microbial communities established in oil-polluted coastal environments. More recently, NGS and “omics” approaches have been applied, allowing to in-depth characterization of microbial communities (Kostka et al., 2011).

These studies allowed to assess the complexity of autochthonous microbial communities related to the oil pollution, revealing *in situ* changes in microbial diversity and their selective response to the presence of oil. Nevertheless, *in situ* approaches have many drawbacks since information access on ecophysiology of oil-degrading microorganisms, their activity and degradation pathways remain still limited.

### EXPERIMENTAL ECOLOGY APPROACHES IN MARINE COASTAL SEDIMENTS STUDIES

Experimental approaches have been developed to address the ecological role of microbial communities on hydrocarbon mitigation in marine sediments. These approaches have progressed as scientific questions arise with the concomitant advances of microbial molecular ecology techniques. Enrichment cultures (or slurries), similar approaches to those used for microbial strains isolation, have been used to tackle the impact of crude oil and petroleum hydrocarbon compounds on bacterial communities. Usually, sediments are mixed with a minimal medium (more often artificial sea water) containing crude oil or selected hydrocarbon compounds, and maintained at laboratory under agitation. Following the dynamic modifications of microbial communities in the slurries, fingerprinting techniques and 16S rRNA gene libraries analyses have shown the ecological succession of microbial populations in response to hydrocarbon compounds in pristine deep marine sediments (Cui et al., 2008), mangrove sediments (dos Santos et al., 2011), polluted harbor marine sediments (Wang and Tam, 2011; Dell’Anno et al., 2012; Rocchetti et al., 2012) and anoxic zone of microbial mats (Abed et al., 2011). While such approaches were useful to estimate the microbial degradation capacities of hydrocarbon compounds (Cui et al., 2008; Abed et al., 2011), the efficiency of bioremediation strategies (Dell’Anno et al., 2012) and the early functional response involved on hydrocarbon degradation (Paisse et al., 2011; Paissé et al., 2012), the simplification of the microbial systems present some limitations. Among them, the destruction of the sediments’ structure imposes a strong limit to extrapolation to complex systems where microorganisms interact each other and with surrounding organisms according to the structure and stratification of sediments. To overcome these limitations, microcosms maintaining intact the sediment structure have been developed. An elegant example is provided by studies undertaken to determine the impact of crude oil on microbial mats (Bordenave et al., 2004, 2007; Lliros et al., 2008). Microbial mats are vertically stratified structures where microbial populations take place according to micro-gradients of oxygen, sulfur and light at the water-sediment interface. Pieces of microbial mats are maintained in microcosms under diel light–dark cycle (16 h/8 h) to ensure the microbial stratification and then exposed to crude oil. Our studies with microbial mats from Camargue (France) demonstrated, by combining T-RFLP and 16S rRNA (gene and transcript) library analyses, the dynamic changes of microbial communities inhabiting microbial mats in response to *Erika* fuel with concomitant degradation of hydrocarbon compounds (Bordenave et al., 2004) and their resilience after 1 year exposure (Bordenave et al., 2007). Lliros et al. (2008) showed the versatility of such microbial communities by demonstrating that microbial communities had distinct behavior according to the type of crude oil using reconstituted microbial mats from the Ebro delta (Spain) maintained under tidal cycles without renewing water. Microbial mats constitute particular ecosystems relatively easy to maintain in microcosms under their initial stratification because they are ecosystems driven by phototrophic microorganisms that impose a selective pressure. In comparison, marine coastal sediments are more susceptible to the fluctuation of environmental parameters due to tide and waves. Thus, the environmental conditions in which they develop are more difficult to simulate. Experimental systems maintaining sediments with tide simulation such as sediment columns (Röling et al., 2002) and sediments maintained in aquarium (Taketani et al., 2010) allowed to determine the role of nutrients bio-stimulation treatments on hydrocarbon-degradation efficiency and the impact of crude oil addition on adapted nitrogen fixation populations, respectively. But, because sediments were homogenized prior microcosms setting, these studies simulate mainly chemical–physical environmental parameters without addressing the impact of the other benthic organisms. In order to further maintain sediments as closer as possible to environmental parameters different strategies have been developed. Katayama et al. (2003) maintaining sediments in a tidal flat simulator, with a wave generator and a tide control device, identified oil-susceptible bacteria as bio-indicator of pollution by combining culture-dependent and molecular approaches. Suárez-Suárez et al. (2011) installed mesocosms *in situ* to assess the role of sulfate reducing bacteria in the degradation of Prestige oil. Similarly, Coulon et al. (2012), maintaining intact cores of coastal mudflat sediments in mesocosms under tidal cycles without renewing water, observed the development of phototrophic biofilm playing a crucial role in hydrocarbon degradation. They also demonstrated the negative effect of oil on the benthic macrofauna that in turn allowed the development of phototrophic biofilm (Chronopoulou et al., 2013). This observation highlights the importance to consider sediments as a whole ecosystem where microbial activities involved on hydrocarbon degradation are driven not only by the presence of contaminant but also by biotic and abiotic factors controlling the microbial web functioning. Appropriate experimental ecology approaches would be useful to decipher the mechanisms determining the organization of microbial communities with efficient hydrocarbon degradation capacities. In the next section we present the experimental approach developed in our lab to address the effect of the reworking activity of the benthic macrofauna (bioturbation) in structuring hydrocarbon-degrading microbial communities and the main results obtained.

## MIMICKING ENVIRONMENTAL CONDITIONS IN EXPERIMENTAL ECOLOGY

In intertidal zones, microbial degradation processes are driven by environmental conditions that are daily modified according to tide level (**Figure 1A**). An original microcosm (**Figure 1B**) system maintaining the structure of muddy sediments under tidal cycles was set up ensuring conditions close to those prevailing in the natural environment of coastal marine sediments (Stauffert et al., 2013a). Coastal marine sediments were sampled with a core collector, and transferred while maintaining their integrity into microcosm boxes. Tidal cycles were applied and natural seawater was renewed with each tidal cycle. The experimental design was drawn with the aim to test the hypothesis that the addition of polychaetes stimulates the bioturbation activity which in turn could select a particular microbial community with an increased biodegradation capability. The conditions applied were: (i) CTRL: control condition, (ii) BAL: oil addition, (iii) NEREIS: addition of *Hediste (Nereis) diversicolor* and (iv) NEREIS+BAL, addition of oil and *H. diversicolor*. Oil contamination was performed on the surface (2 cm top layer) after homogenization with sediments (BAL and NEREIS+BAL). *H. diversicolor* was added to the microcosms in order to increase sediments reworking (NEREIS and NEREIS+BAL). By following, over a 9-month period, the petroleum removal, the macrofaunal reworking activity and the microbial communities’ structures and compositions, we demonstrated that the modification of the microbial community structure in mudflat sediments after petroleum addition was dependent on the presence of the added burrowing polychaetes *H. diversicolor* (Stauffert et al., 2013a, b). Contrary to our initial hypothesis, despite that the addition of burrowing organisms stimulated the bioturbation activity and modified the microbial community structure, the overall oil removal capacity was not affected by the addition of polychaetes. For Bacteria (**Figure 1C**), although both BAL and NEREIS+BAL communities were dominated by Gamma- and Delta-proteobacteria, important differences were observed at the genus level. The BAL community showed more diversity with the presence of minor phyla and a slight increase of Alpha-proteobacteria (Stauffert et al., 2013a). For Archaea (**Figure 1B**), BAL community was represented only by Crenarchaeota MCG members while NEREIS+BAL community exhibited more diversity (Stauffert et al., 2013b). Thus, from an initial microbial community two distinct communities showing a similar overall oil removal capacity were obtained. By adding burrowing organisms to sediments maintained near-environmental conditions we were able to manipulate microbial community structure and composition, opening the way for the study of the mechanisms underlying microbial community restructuring after environmental perturbations which includes resistance, resilience, and functional redundancy (Allison and Martiny, 2008). Combining metagenomic and metatranscriptomic analyses with metabolite profiling will provide valuable information to understand the mechanisms underpinning the bacterial communities structuring, particularly the role of the rare microbial members in the establishment of structure/function relationship. Such approach has demonstrated the influence of hydrocarbon compounds on the microbial community inhabiting the deep-sea sediments of the Gulf of Mexico after DH oil spill (Kimes et al., 2013). The application of correlation and co-occurrence analyzes from metagenomics and 16S bar-coding profiling, that allows to forecast microbial interactions and metabolic networks (Faust and Raes, 2012), to experimental systems may offer the possibility to gain in depth information on how microbial communities behave after a disturbance (Shade et al., 2010; Knight et al., 2012) and define a “core” community ensuring the basal ecosystem functioning. Experimental systems authorize comparative metatranscriptomics approaches (Bordenave et al., 2009, 2010) that combined with high-throughput sequencing can provide the discovery of novel genes expressed during phytoplankton bloom with the possibility to explore the structure/function relationships (Gilbert et al., 2008). Another example of metatranscriptomic analysis is the identification of a set of genes, including hydrocarbon degradation, stress response and detoxification genes, induced after the environmental disturbance by phenanthrene in soil microcosms (de Menezes et al., 2012). The structure/function relationship can be further elucidated by metaproteomics, enabling the identification of proteins present in the community. The metaproteogenomic approach, that combine metagenomic and metaproteomic analyzes, allowed to describe the metabolism related to naphthalene degradation in soil by comparing four microbial communities maintained in microcosms (Guazzaroni et al., 2013). However, metaproteomics needs further development involving mass spectrometers with high sensitivity to access low abundant proteins (Zarraonaindia et al., 2013). Our microcosm system allows to propose an experimental ecology approach to determine how the fluctuation of environmental parameters, particularly oxygenation and redox oscillations resulting from the biological (bio-turbation) or mechanical (physical-turbation) reworking of the sediment, influence the coupling between bacterial functional groups and their degradation capacities.

## CONCLUDING REMARKS

**FIGURE 2:**
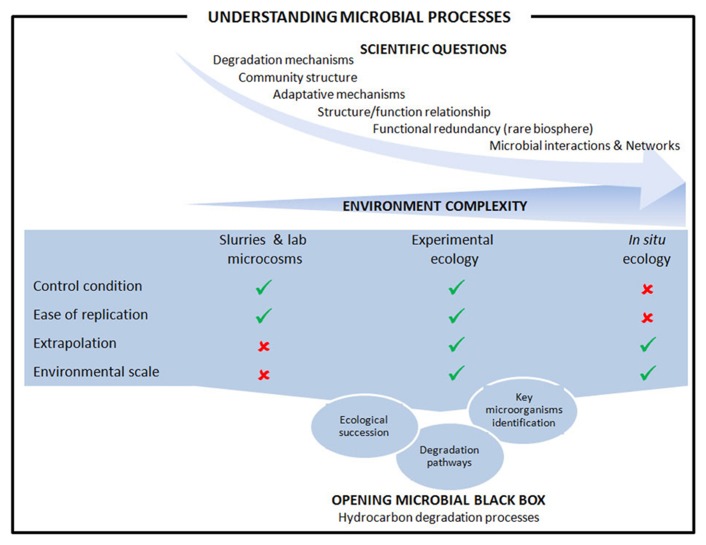
**Understanding microbial processes involved on hydrocarbon degradation in marine coastal sediments.** The general scheme from scientific questions to microbial processes is presented together with the comparison of experimental approaches developed at different environmental complexity.

We reviewed here recent approaches implemented in order to assess microbial processes involved on hydrocarbon degradation in marine coastal sediments. Microbiologists have developed several approaches, including more or less sophisticated experimental systems and *in situ* studies to answer the scientific questions regarding the microbial mechanisms that take place in response to oil and hydrocarbon contaminations. **Figure 2** summarize the main advantage and limitations for the approach considered in addressing the scientific questions. Experimental ecology using experimental systems mimicking as close as possible the environmental conditions combine the advantages of lab controlled systems with the possibility of extrapolation to the real situation found in complex ecosystems. Such approaches offer the opportunity to conduct experiments in replicates, crucial advantage for robust statistical analyses as highlighted by Prosser (2010). The advent of next generation sequencing technologies combined with high-throughput methods assessing functionality (proteomics and metabolomics) has allowed the development of systems biology, a holistic approach to understand complex biological systems. However, because the sediments’ ecology in coastal areas is extremely complex, analysis using system biology tools at different environmental scales would be useful to elucidate microbial hydrocarbon degradation processes.

## Conflict of Interest Statement

The authors declare that the research was conducted in the absence of any commercial or financial relationships that could be construed as a potential conflict of interest.
